# Factors Important to the Prioritization and Development of Successful Topical Microbicides for HIV-1

**DOI:** 10.1155/2012/781305

**Published:** 2012-07-12

**Authors:** Karen W. Buckheit, Robert W. Buckheit

**Affiliations:** Topical Microbicide and STI Research Department, ImQuest BioSciences, Inc., 7340 Executive Way, Suite R, Frederick, MD 21704, USA

## Abstract

Significant advancements in topical microbicide development have occurred since the prevention strategy was first described as a means to inhibit the sexual transmission of HIV-1. The lack of clinical efficacy of the first generation microbicide products has focused development attention on specific antiretroviral agents, and these agents have proven partially successful in human clinical trials. With greater understanding of vaginal and rectal virus infection, replication, and dissemination, better microbicide products and delivery strategies should result in products with enhanced potency. However, a variety of development gaps exist which relate to product dosing, formulation and delivery, and pharmacokinetics and pharmacodynamics which must be better understood in order to prioritize microbicide products for clinical development. *In vitro, ex vivo*, and *in vivo* models must be optimized with regard to these development gaps in order to put the right product at the right place, at the right time, and at the right concentration for effective inhibition of virus transmission. As the microbicide field continues to evolve, we must harness the knowledge gained from unsuccessful and successful clinical trials and development programs to continuously enhance our preclinical development algorithms.

## 1. Introduction

Significant progress has been made in the development of topical anti-HIV microbicides since their initial description and development nearly 20 years ago. The first products developed for microbicide use were nonspecific agents which prevented HIV-1 from entering target cells by disrupting the viral membrane, including nonoxynol-9 (N-9), SAVVY (C31G), and Ushercell (cellulose sulphate) [1–3]. Clinical results with N-9 demonstrated enhanced rates of infection in the treated groups, suggesting the surfactant caused vaginal damage which allowed greater rates of infection [3]. SAVVY was prematurely discontinued due to the HIV incidence being half of the expected rate (one of the characteristics rendering the trial uninformative) [1]; however, it could not be conclusively determined that SAVVY promoted HIV infection as in the case of N-9. Ushercell was also discontinued due to a higher rate of infection compared to the placebo group [2]. Following the failure of the nonspecific surfactants, microbicide development has focused on the identification and development of specific antiretroviral (ARV) agents targeted at preventing early steps in virus replication such as virus attachment and entry and reverse transcription. Most recently, microbicide development has expanded to include the evaluation and development of late-acting products (integrase and protease inhibitors) [4], agents directed at cellular targets important to virus replication and transmission, and agents which boost mucosal and innate immunity to HIV [5]. The first specific antiretroviral compound evaluated was PRO2000, a synthetic naphthalene sulphonate, which specifically targeted CD4 to prevent virus attachment and subsequent entry. PRO2000 was proven safe in Phase I/II human clinical trials but eventually was shown to be ineffective in preventing HIV transmission [6]. In 2010, results from the CAPRISA 004 Phase IIb trial demonstrated that a 1% tenofovir gel reduced HIV transmission by 39% in the study population overall and by 54% in women with high levels of adherence to the study protocol [7]. The CAPRISA 004 study provided the first positive results which demonstrated that an antiretroviral agent formulated as a vaginal gel could successfully prevent the sexual transmission of HIV, energizing the field of microbicide development. Unfortunately, in the latest clinical trial (VOICE) performed by the Microbicide Trials Network (MTN), equivalent numbers of infections were observed among women in both the placebo and 1% tenofovir gel arms, and the trial was subsequently discontinued [8]. Differences in dosing regimens between the CAPRISA 004 and VOICE trials have been suggested to have contributed to the different results of these trials. The ongoing FACTS trial being performed in South Africa is set to address the reproducibility of pericoital dosing with tenofovir gel. Although the results of VOICE were a disappointment to the microbicide community, the lessons learned and information gained from the tenofovir trials (CAPRISA and VOICE) as well as the other failed trials may prove to be informative and enable the field to better optimize and develop an efficacious microbicide.

Microbicide efficacy clinical trials are very large trials, enrolling anywhere from 800 to over 9,000 women in each trial [9]. Thus, to have multiple failed trials of this magnitude has been extremely costly from both a financial and human life perspective. The risk of trial failure is uniquely high due to the limitations of Phase II trials. Although these trials often provide go-no-go indication, they are very unlikely to have sufficient power to report anything relative to efficacy due to the low seroincidence rates, even in high-risk populations. Since the key to the identification and development of a successful microbicide product likely lies in the performance of better and more informative preclinical evaluations, a greater understanding of the optimal delivery and the pharmacokinetic and pharmacodynamic (PK/PD) profiles of both the active pharmaceutical ingredient (API) and the formulated clinical product is necessary prior to the initiation of human clinical trials. Two decades of research and development and the outcomes of successful and failed human clinical trials have served to define a variety of gaps in the preclinical microbicide development pathway. Thus, as the microbicide development field moves forward to define new products and design informative and successful clinical trials, it is critical to understand how these development gaps might be effectively filled in order to generate the data necessary to understand how to best optimize and prioritize microbicide product development. These critical topical microbicide development gaps may be defined as follows: (1) a better understanding of the environment in which the microbicide must act, including the positive and negative effects of the presence of semen and vaginal fluids, natural and pathogenic organisms, and the physiology of the biological compartments (vagina and rectum); (2) a better understanding of the pharmacokinetic and pharmacodynamic properties of the microbicide product and the use of *in vitro*, *ex vivo,* and *in vivo* models to quantify these critical candidate product properties; (3) a better understanding of means to define appropriate dosing concentrations of a microbicide product and how the dose, formulation, and delivery vehicle impact the pharmacokinetic and pharmacodynamic properties of the product; (4) a better understanding of critical issues in the formulation and delivery of the microbicide products for use in both the vagina and rectum, including the use of dual compartment and oral dosing strategies; (5) the effective implementation of multipurpose prevention technologies, involving broad based anti-infective and contraceptive products. Inherent in each of these development gaps is the overarching goal of developing a product which is acceptable to the population of individuals that will primarily use the products that are developed. Herein we will evaluate each of these development gaps and discuss how information obtained during preclinical development might be improved and better utilized to identify and prioritize microbicide products for development. The most critical requirement of the microbicide development algorithm is the need to have the *right concentration* of the *right microbicide* (or combination of microbicides) present at the *right location* and at the *right time* to prevent HIV infection. Thus, the microbicide development gaps require a more intimate understanding of dosing, formulation, and delivery vehicles, which will result in effective pharmacokinetic and pharmacodynamic properties for candidate products, and allow the right products to be prioritized for development. Since each microbicide is unique, these variables must be independently evaluated in order to develop highly effective microbicide products.

## 2. The Role of the Complex Biological Environment

The biological environment in which an active microbicide product must act has come to be recognized as a critical variable to microbicide functionality. Microbicide products are now being developed for both vaginal and rectal use and therefore the anatomy and physiology of both compartments needs to be considered as a key feature of the preclinical development algorithm. The complexity of the vaginal environment includes the anatomical features of the cavity, the presence of naturally occurring and pathogenic microorganisms, and the presence of vaginal fluids and mucus. The environment becomes even more complex with the act of coitus and the deposition of semen into the vault. The vagina possesses its own inherent defense mechanisms including the multilayered squamous epithelium which acts as a natural barrier to infection, the hydrogen peroxide producing vaginal flora (*Lactobacillus*) which maintains an acidic environmental pH, mucus which provides a physical barrier to virus transport, and the production of a variety of antimicrobial and innate defense molecules which directly and indirectly inactivate virus or suppress infection and virus replication. It is important that the integrity of this environment be maintained as the first line of defense against HIV infection; the development of all microbicide products involves the early evaluation of the effects of a candidate product on the components of this primary defensive barrier. Microbicide products should be nontoxic to the cellular and tissue structure, should not result in the elimination of the normal protective populations of H_2_O_2_ producing *Lactobacillus*, and should remain stable and active at low pH (approximately pH 4.6). All of these properties of a topical microbicide candidate can be accurately assessed in preclinical *in vitro* assays [10, 11]. Maintaining normal vaginal fluids and mucus is also important given that these products typically act as the initial line of defense against infection by microorganisms and HIV [12, 13]. In addition to innate immune responses and microbe-sensing properties, including the production of antimicrobial peptides [14, 15] and proinflammatory cytokines, cell-free and cell-associated viruses are also inactivated at low pH [16–18], and movement to potential target cells is restricted by vaginal mucus. Anti-HIV activity has been directly attributed to components of vaginal fluids, including defensins [19, 20], toll-like receptor (TLR) agonists [21, 22], and secretory leukocyte protease inhibitor (SLPI) [23]. Many studies have been performed to evaluate the antiviral effects of cervicovaginal fluid (CVF). Ghosh et al. showed that CVF incubated with virus prior to the addition of target cells yielded 0 to 100% inhibition of infection, with some samples showing enhancement of virus infection. This study concluded that a wide range of factors that are capable of mediating antimicrobial protection are present in CVF and specifically correlated levels of HBD2, MIP 3*α*, and HIV-specific IgG antibodies with the protection of target cells from infection with HIV [24]. Other studies have served to confirm and expand these results demonstrating the HIV-inhibitory activity of CVF, and laboratory investigations continue to better understand and harness these protective effects of CVF [25–27]. The results of these experiments suggest pluripotent antiviral effects exerted by a variety of CVF constituents working in concert as opposed to the individual activity of any single product results in the natural inhibitory potential of CVF. Therefore, it is critically important that a microbicide product should not diminish the natural protective effects of vaginal fluid, and all products should be evaluated *in vitro* and *ex vivo* in the presence of CVF to verify that biological activity is maintained.

Another important consideration in the context of vaginal fluid involves the spread, coverage, and dispersion of the microbicide product during sexual intercourse. In studies performed by Keller et al., CVF collected by lavage within an hour following a single dose of 0.5% PRO2000 gel significantly inhibited HIV when evaluated in *in vitro* antiviral assays. This antiviral activity was significantly reduced when the CVF was collected following sexual intercourse, and no significant protective effect was observed in postcoital CVF obtained in the presence compared with the absence of PRO2000 gel application [28]. These results suggest the physical act of sexual intercourse results in mixing and dispersal of the microbicide product resulting in reduced effectiveness of a topical microbicide, and these factors should be evaluated during early product development. Instrumentation and methodology to perform studies to evaluate gel spreading and overall epithelium coverage within the vagina and rectum have been developed and employed to evaluate microbicide products in the context of coitus, and these evaluations will help to determine if a microbicide product is able to be in the right place and right concentration to prevent infection prior to and following coital events [29–31]. Confocal Raman spectroscopy (CRS) has recently been utilized to measure local concentrations of APIs in three dimensions in vaginal or rectal fluids, gels, and tissue explants, and this methodology may yield highly relevant data regarding the penetration of API into vaginal and rectal tissue [32].

Over the past two decades the primary focus of microbicide development has been on preventing vaginal HIV-1 transmission. However, in the developed world unprotective receptive anal intercourse (URAI) is the primary risk factor for HIV acquisition in the MSM population. URAI is now recognized as a significant feature of the sexual practices of women in both the developed and developing countries of the world [33, 34]. The vulnerability of the fragile intestinal mucosa to HIV transmission yields a 20-fold greater infection risk per sex act compared to the infection risk from unprotected vaginal intercourse. Furthermore, the rectum, unlike the vagina, is an open ended, fragile, and poor barrier to pathogens, resulting in an increased risk of infection during URAI. The mucosa accounts for approximately 10% of the colorectal wall thickness and is comprised of single layered epithelium, lamina propria, and muscularis mucosa. As with vaginal virus transmission, it is thought that virus migrates through the epithelial cell layer to the lamina propria where a greater frequency of target cells is present and primed for infection. The gut mucosa comprises the bodies' greatest reservoir of CD4+ cells and other immune competent cells. Ninety percent (90%) of colonic CD4+ cells express the HIV-1 chemokine coreceptor CCR5, rendering this environment a vast reservoir of target cells for HIV-1 infection and transmission. Upon establishment of sites of infection, the presence of an adequate local density of activated target cells for local amplification of the virus and subsequent dissemination to the systemic circulation is required and the gut mucosa appears to provide this susceptible environment to the virus [35]. The fragile nature of the rectum makes it more susceptible to tears and damage during receptive anal intercourse (RAI) which also promotes infection. As with the vagina, infection of the rectum by other opportunistic microorganisms can also increase susceptibility to HIV-1 infection. Sixty percent (60%) of HIV-negative men have been shown to be positive for anal human papilloma virus (HPV), and this number increases to 95% in the HIV-positive male population [36]. Smith et al. reported an increased risk of HIV acquisition among Kenyan men infected with HPV which may derive from the lesions associated with HPV infection, as observed in women with HPV infection [37]. Although some similarities exist between the vaginal and rectal compartments, significant differences in anatomy and physiology exist, and these differences need to be taken into account early in topical microbicide product development. 

**Figure 1:**
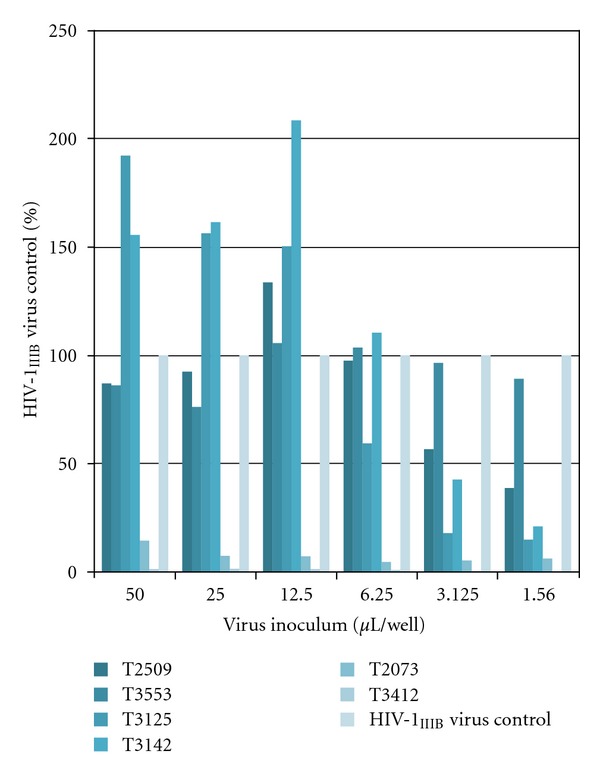
Biological impact of semen on virus infectivity and replication. Fifty samples of whole semen (Lee Biosolutions) were evaluated for biological activity in HeLa-CD4-LTR-*β*-galactosidase cells infected with varying quantities of infectious HIV-1_IIIB_. Representative results obtained with six of these samples are presented. Semen was added to the cells in a volume of 50 *μ*L immediately prior to the addition of infectious virus at 6 different virus inoculums ranging from a high inoculum of 50 *μ*L (straight virus) and five additional serial twofold dilutions of virus in tissue culture medium. At 4 hours the virus and semen were washed from the monolayer of cells, and the cultures were incubated for an additional 48 hours at which time virus replication was quantified by *β*-galactosidase production in the cultures. The results presented demonstrate the three patterns of biological activity observed among the 50 tested samples: (1) enhanced levels of infection (see samples T3142 and T3125 at high virus inoculum), (2) inhibition of infection (T2073 and T3412 as well as T3125 and T3142 at lower viral inoculum), and (3) no effect on infection (T3553).

As mentioned above, the complex environment of the vagina and rectum becomes even more complex upon the deposition of semen. When semen is introduced into the vaginal environment a variety of changes occur. The first and most significant effect is that semen changes the acidic vaginal pH to near neutral pH which alters the balance of normal flora and provides an environment which facilitates the rise of bacterial vaginosis and yeast infections. Additionally, semen deposited into the reproductive tract promotes an influx of activated inflammatory cells in close proximity to infectious virus and virus-infected cells in the semen [38, 39] and induces changes in the population of leukocytes which are present in the vaginal tract (reviewed in [40]). Semen also can have a “toxic” effect to the vaginal environment that results in recurrent vaginitis that is associated with localized irritation and inflammation [41]. This inflammatory reaction yields additional recruitment and activation of HIV-1 target cells which ultimately facilitates HIV infection and transmission and sometimes results in enhanced HIV-1 infection. The enhanced infection effects can be attributed to the neutralization of the acidic vaginal pH promoting the survival of cell-free and cell-associated virus, the presence of semen-derived enhancer of virus infection (SEVI), and mediation of the electrostatic interaction of spermatozoa with HIV-1 virions (reviewed in [40]). Further, studies performed by Lai et al. have shown that the neutralization of vaginal pH by semen increases the movement of HIV virions in mucus possibly resulting in infectious virus more rapidly reaching the epithelium [42–44]. However, semen has also been shown to possess antiviral properties. The inhibition has been experimentally attributed to the oxidation of SP polyamines by diamine oxidase in the vaginal environment producing radicals that inactivate HIV, cationic polypeptides that are contained in seminal plasma, and the interference of the attachment of HIV-1 to DC-SIGN by a potent inhibitor contained in seminal plasma (reviewed in [40]). In this regard, we have evaluated the antiviral activity of 50 individual semen samples obtained from individual donors, and we have shown both inhibition and enhancement of HIV infection mediated by these diverse semen samples (Figure 1). In the rectum, semen has similar effects as those observed in the vagina. Once a trauma-inducing event occurs, the inflammatory cytokines enable transmission of virus through the epithelial barrier. Thus, current research supports the fact that microbicide candidates must be evaluated in the presence of semen to verify the potency of the candidate and to confirm that there is no antagonism of antiviral efficacy or enhanced toxicity. As was discussed with vaginal fluids, it has also been shown that semen provides a physical barrier to the movement of virus from the semen towards target cells in the epithelium of the vagina or rectum [45, 46].

In both the vagina and the rectum, the form in which infectious virus is presented in these environments must also be carefully considered (cell-free virus versus cell-associated virus, as well as combinations of both forms) for optimal development of a microbicide since cell-associated virus may be less susceptible to some microbicide candidates compared to cell-free virus. Louissaint et al. have recently reported that when using surrogates for cell-free and cell-associated HIV and semen, cell-free and cell-associated surrogate distribution following simulated intercourse coincided within the female reproductive tract [47]. In a small group of gay men (6 total), Butler et al. showed that the virus being transmitted was more closely related to the free virus in seminal plasma [48]. This would, however, need to be confirmed in a larger study. A better understanding of the roles of cell-free and cell-associated virus in establishing infections in the vagina and rectum remains controversial and should continue to be investigated.

## 3. Pharmacokinetics and Pharmacodynamics of Microbicide Products

Over the past several years the importance of pharmacokinetics (measurement of microbicide distribution, absorption, and retention typically measured in tissues and body fluids) and pharmacodynamics (microbicide biological activity within the compartment) has been increasingly recognized within the microbicide community as a crucial feature in understanding microbicide efficacy and toxicity. *In vitro* and *ex vivo* assays have been designed to better understand these parameters prior to the introduction of products to human clinical trials. Understanding effective API pharmacokinetics also requires an understanding of the mechanisms of HIV infection of vaginal or colorectal tissues and subsequent dissemination of the virus from the initial sites of infection. In nonhuman primate models, 30-to-60 minutes of exposure to an infectious inoculum are sufficient to establish a productive and spreading virus infection [49]. Transmitted or founder viruses target CD4 populations in the mucosa that express high levels of the CCR5 chemokine coreceptor [50]. In explant and NHP models, virus has been demonstrated to penetrate the superficial layers of the stratified epithelium which enables the virus to quickly come into contact with T cells and Langerhans cells contained within these surfaces [35]. Additional NHP studies have demonstrated that the initial infection of cells in the mucosal surfaces occurs within 16 to 72 hours and an established and spreading infection from these sites requires an influx of additional activated T cells [35]. A microbicide will only be effective if the product is able to either prevent the virus from infecting these critical target cell populations or is able to effectively prevent the establishment of a spreading infection from these initially infected cell foci. For this reason pharmacokinetic (PK) and pharmacodynamic (PD) assessments need to be performed and understood in terms of the cells and tissues which must be protected by the microbicide (i.e., epithelium versus stroma, introitus versus cervical os, etc.).

Historically, PK measurements were performed as a component of microbicide safety studies as well as to quantitatively determine if vaginal or rectal delivery resulted in significant API absorption to the systemic circulation. PK/PD evaluations have been routinely performed in animal models which include nonhuman primates, humanized mice, and sheep (reviewed in [51]). Rectal PK studies have been routinely performed in pigtailed macaques [52]. Thus, to date, PK assessments have not been routinely utilized to facilitate determination of the required effective dose of a formulated microbicide product that would be optimized for delivery or to even confirm if sufficient API can be delivered to the critical tissues where infection occurs but have been used to confirm that the dose being used is nontoxic and safe. Based upon the results of recent clinical trials such as CAPRISA 004 and VOICE, it has become apparent that PK/PD studies need to be performed to better understand where API goes in the cells, tissues, and fluids of the vagina and rectum, at what concentrations the API is found in both fluids and relevant infectable tissue, and if sufficient API is present to actually interfere with the infection of HIV.

Current PD evaluations for microbicides involve *ex vivo* infection inhibition studies using tissues from microbicide-treated animals. Multiple biopsy tissues from treated and control untreated animals (or from human volunteers in clinical studies) are exposed to infectious HIV, and the ability of HIV to infect and replicate in the tissue is quantified. Although these studies provide important information and help to bridge the gap between *ex vivo* and *in vivo* evaluations, it is not yet clear if this methodology will accurately quantify the effectiveness of a microbicide in human clinical trials. Similar studies using vaginal lavage to determine if protective concentrations of microbicide products are achieved in vaginal fluids are also being utilized to monitor the attainment of effective microbicide concentration levels [28].

Although nonhuman primate models have been the animal model of choice for PK/PD evaluations and have provided great insight into HIV transmission in the vagina and rectum, they have not proven to be the best predictive model for efficacy of a microbicide product in human clinical trials. For example, whereas complete protection of macaques was achieved with the 6% cellulose sulphate gel, no protection was observed in human volunteers in the clinic [53]. With a 1% tenofovir gel, efficacy studies in nonhuman primates demonstrated protection in all animals whereas in the clinical trial only 39% of women were protected [54]. A topical microbicide development model using mice has also been reported [55, 56] employing humanized bone-marrow-liver-thymus (BLT) mice reconstituted with human CD4+ T and other relevant human cells which are susceptible to intravaginal infection by HIV-1. However, due to the nature of the samples required for evaluations, PK/PD studies are often difficult to perform with the mouse model. Within the microbicide field, consensus has not been achieved on the predictive value of animal models, and many development programs forgo animal efficacy studies in favor of Phase 1 human studies.

For this reason, more predictive and robust and less expensive PK/PD models need to be established for evaluation of products prior to human clinical trials. The *ex vivo* cervical explant model has been extensively used for the evaluation of microbicide safety (toxicity assays) and is currently being utilized for PD evaluations using explant tissue from both human and animal studies. It had been assumed that the evaluation of microbicide efficacy and toxicity in human cervical explant tissue would provide highly relevant data to bridge the gap between *in vitro* results and *in vivo* efficacy evaluations, given the explants are more representative of the tissue and cells being targeted in the vagina (epithelial and immune target cells). In addition, the explant studies are much less expensive than the nonhuman primate models. Unfortunately, data obtained from cervical explant evaluations suggests that the model may not yield conclusive evidence of product efficacy, and there are varying schools of thought on whether these explant studies actually provide added and reliable information beyond that obtained from *in vitro* systems or in animal modeling studies. Besides variability in protocols utilized to perform the explant assay [57–59], there are a variety of limitations to the use of cervical explant cultures, including lack of hormone modulation, lack of recruitment of immune cells, loss of epithelium, and the inability of the explant tissue to regenerate/repair itself [58]. Variability in culture conditions and the relative ability of HIV to grow in the explant cultures, with significant background attributed to bound but nonreplicating virus, confound the interpretation of explant results. Infectious virus has been shown to replicate in the explant cultures, however the cell population where virus is detected can change. Although significant issues with data interpretation exist, Beer et al. have correlated their explant data to both animal studies [60, 61] and to safety and acceptability trials [61–65] validating the use of cervical explants in microbicide development. As with any of the *in vitro* and *ex vivo* models that have been developed, the use of cervical explants will only be completely validated when the data is correlated to that which was observed in the human clinical trials. Continuing evolution of the explant models will also result in significant enhancement of the utility of the evaluations.

Although cervical explant evaluations may prove to be a needed and predictive component of the microbicide development algorithm, the limitations suggest that additional development must occur to provide more relevant *in vitro* models to understand how a microbicide will function in humans. One of the model systems now being used in the microbicide development community involves *in vitro* evaluation of drug permeability and transport across epithelial cell monolayers. These models evaluate the ability of a microbicide product to transit from the delivery vehicle (gels, rings, films, etc.) and across cell/tissue barriers representative of epithelia cell layers that would be present in the vagina and rectum. These studies are performed in a two-compartment Franz cell apparatus with appropriate tissue culture cells and/or ectocervical tissue [66]. Following incubation of the microbicide product with the cells and/or tissue, sample analysis to quantify microbicide product content is performed utilizing high-pressure liquid chromatography (HPLC). Using these models, Rohan et al. found that tenofovir from a 1% gel permeates into the tissue but the quantity of tenofovir measured after 30 minutes differed among individual ectocervical samples [67]. Mesquita et al. have developed an *in vitro* assay using a transwell assay system that can evaluate both the safety of a microbicide as well as the PD properties of a microbicide [68]. In these assays, a microbicide product must transport through an epithelial cell barrier to the lower tissue culture chamber where the product must protect target cells from *in vitro* infection by infectious HIV-1. Toxicity to the barrier cells can be measured by transepithelial resistance (TER). These *in vitro* assays allow for the evaluation of multiple microbicides at many concentrations. Although not yet proven, these *in vitro* assays may provide relevant information that will help prioritize microbicide candidates for clinical development. 

## 4. Microbicide Dosing and Its Critical Impact on Pharmacokinetics, Pharmacodynamics, and Clinical Efficacy

The initial identification and subsequent development of a successful microbicide are dependent on the robustness of the efficacy and safety testing algorithms that are used to advance products. Preclinical and clinical experiences have driven the natural evolution of these algorithms over time, and it is understood that the algorithms will continue to change in the future [10, 11]. These algorithms should be capable of comparatively evaluating the active pharmaceutical ingredient (API) and the final formulated product with appropriate control compounds, as well as other experimental and approved microbicide products. One of the more difficult parameters to be addressed as a microbicide developer involves the quantitative determination of the clinical dosing of the product. Consensus opinion from nonefficacious and successful development programs and trials suggests that “*more is better*” and to “*dose as high as possible*” to assure that an effective concentration of API is present where and when it is needed to prevent virus transmission. This method of defining the API dosing in the final formulated product may result in a dramatic underestimate or overestimate of the amount of API that is actually required (or achievable), resulting in the extremes of lack of efficacy or potential safety issues. *Ex vivo* and *in vivo* evaluations in monkeys and mice have provided some information on the permeability/uptake of API into tissues in order to better understand the pharmacokinetics and pharmacodynamics properties of the compound and how they may relate to dosing levels; however, the tissue concentrations achieved with high dosing levels of a microbicide are significantly higher than the inhibitory concentrations achieved using cell-based *in vitro* assays. In light of the type of cells used in the *in vitro* evaluations, the lack of robustness of the assays in terms of their quantitative endpoints and timing of endpoint analysis, and the importance of understanding dosing and the prioritization of compounds for clinical use, it is critically important to understand the dosing requirements of an API as early as possible in the development process. Additionally, the differences observed between *in vitro* and *in vivo* effective levels could be attributed to poor distribution, that is, failure to coat all of the folded surfaces. The recently reported microbicide transmission and sterilization assay (MTSA) may provide a quantitative *in vitro* model to predict the tissue API concentration required to prevent virus transmission, and these data may then determine the required dose concentrations of the microbicide product to achieve that tissue API level [69].

The MTSA serves to define the concentration of a microbicide product required to completely suppress the transmission and subsequent replication of transmitted viruses in culture, yielding sterilization of HIV from a culture of cells [69, 70]. In the MTSA, virus is added to the culture in a cell-free or cell-associated form, and the virus infection is allowed to proceed over the course of serial passaging of the infected cells in the presence of various fixed concentrations of the microbicide test compound. The cells are subcultured every three days by adding 20% of the infected culture (cells plus supernatant) to the same original volume of uninfected cells in fresh medium with the same fixed concentration of test agent. At each passage, the cultures are evaluated for virus replication in the culture in order to quantify the timing of virus breakthrough (or frequency of infected cells) at each compound concentration. The concentration at which the compound totally suppresses virus replication in the culture is defined as its sterilizing concentration, and this sterilizing concentration is unique for each microbicide product we have evaluated and in most cases is significantly higher than the 50% inhibitory concentrations defined in the shorter term and less robust standard transmission inhibition assays most typically employed for microbicide development (Table 1). With more potent inhibitors, the sterilizing concentration may correlate with the 99% inhibitory concentration of a product in the standard inhibition assays. Thus, the MTSA can be utilized to understand how much of an API will be necessary at the site of infection in order to totally suppress virus infection and replication and to prioritize a panel of APIs for clinical development. The MTSA can be miniaturized and the assay duration minimized by utilization of highly sensitive means to determine the amount of virus present in the culture after infection in the presence or absence of the microbicide product (Q-RT-PCR endpoint) or by quantitatively measuring the number of infected cells in the culture. There are, however, limitations to the MTSA which include the inability of the assay to appropriately mimic the complexity of the *in vivo* situation and the current lack of correlative data with clinical trials that have been successful.

It is possible that dosing determination according to the principles of “*more is better*” and “*go as high as you can go*” will not yield the most effective strategy for defining the dose of a microbicide product for product development. With multiple microbicide trials showing lack of efficacy of the potential microbicide products, it is necessary that we understand if the product failure was a function of lack of potency, if the API was not where it needed to be at the right inhibitory concentration, or if API or excipient toxicities could have led to failure by increasing susceptibility, mediated by reduction barrier effectiveness, or by increasing target cells or receptor density. Critical to all three of these potential explanations is how the microbicide is formulated and delivered. First generation microbicides were developed as coitus-dependent gels. Although this delivery mechanism may be useful in some communities, it may prove impractical in developing countries where a woman might not know when she is going to have sexual intercourse, and societal norms are not accepting of microbicide use. Adherence has been an issue in clinical trials utilizing this dosing strategy [71] and may explain the divergent results of the recent tenofovir trials. Based on the successful use of nevirapine to prevent mother-to-child transmission, the CAPRISA 004 trial was designed so that women would use the tenofovir gel within 12 hours prior to having sex and within 12 hours after having sex (BAT24). Although the relative contribution of each gel application to protection from virus transmission is unclear, it was the first trial to demonstrate marginal protection from infection by HIV [7]. 

Daily dosing is a third strategy to formulate and administer a microbicide. In the VOICE trial subjects were asked to apply the microbicide once a day independent of sexual intercourse [72]. It was hoped that adherence to the regimen would promote a steady-state drug level and that there would be a high adherence rate since administration of the product would become a daily routine, similar to that of oral contraceptives. The vaginal gel arm of the trial was prematurely discontinued because there was no difference in effect demonstrated between the drug-containing gel and a placebo gel [73]. As of the time of this publication, it is not known if the lack of effect was due to lack of adherence to the protocol design. Daily dosing may also prove to be an “inconvenience” to women who are having infrequent intercourse which may actually diminish adherence to the microbicide [74].

A fourth dosing strategy is sustained microbicide delivery through an intravaginal ring (IVR). Based on the successful use of hormonal contraception rings, microbicide rings can be worn safely for up to a month and have already proven they can deliver drug over that period of time [75, 76]. Although IVRs are designed to deliver optimal concentrations of drug for protection, they may not release drug equal to the amount of drug being released from daily dosed and coitally dependent gels and films. IVRs do address issues of coitus independence and long-term dosing strategies which have been issues of significant research focus in the microbicide community for the past decade.

With the proven concept that the optimal formulation of a microbicide product will assist and promote the uptake/permeability of an API through the epithelium and into the vaginal and rectal mucosa, the mechanisms by which this API facilitation occurs need to studied and monitored to best take advantage of optimization of formulation design. We have shown that the uptake of pyrimidinedione microbicide products is critically dependent on the appropriate formulation of the API (unpublished data). A better understanding of the role of the formulation and delivery mechanisms thus is critically important with regard to achieving adequate fluid and tissue PK/PD and defining the required dose of the microbicide product to deliver the API at the right concentration to the target cells.

As we continue to understand virus transmission and dissemination through the mucosa, better formulations and delivery vehicles can be developed which will in turn allow us to better evaluate where formulated products are delivering API and at what concentration. This will be important in the design of a product that is deemed acceptable to the end user.

## 5. Development and Formulation of Microbicides for Dual Compartment Use

As mentioned previously, URAI is one of the highest-risk sexual behaviors for HIV-1 transmission—10 to 20 times riskier than unprotected vaginal sex [34, 77]. In addition, RAI is a component of the sexual practices of both men and women, and among women receptive anal and vaginal intercourse often occur within the same sexual encounter. Additionally, there is increasing evidence that unprotected RAI is being practiced at greater frequencies than previously appreciated by both women and men, in both the developing [33, 78] and developed [79] world. Therefore, there is a real need to develop microbicide products to be delivered rectally as an integral part of the HIV prevention portfolio. Since the practice of URAI is not limited to men who have sex with men (MSM), microbicide products suitable for both rectal and vaginal application are highly needed. Use of a single microbicide product that is safe and efficacious for both vaginal and rectal use would thus be much more convenient (as well as safe and acceptable) than the use of two separate products. Furthermore, use of a single, specifically developed dual compartment product would likely be much more protective than improper utilization of a vaginal microbicide in the rectum, which could potentially increase virus transmission or result in significant safety issues. Finally, rectal microbicides are promising in that little behavior modification would be required to add microbicide protection since lubrication is already a common practice with RAI.

There are some profound differences in the vaginal and rectal compartments that warrant the use of differently formulated products for each. Several safety studies have been performed evaluating the toxicity of vaginal gels in the rectal compartment [80–82]. The results of these studies led to the design of microbicides specifically for rectal administration. One of the key differences in the design of these gels is that vaginal microbicides tend to be hyperosmolar resulting in a gel that is more concentrated than body fluid and ultimately one that will lead to rectal mucosal damage as they will swell with rectal application [78]. Rectal microbicides will need to be isoosmolar to circumvent this potential problem. Additionally the surface area requiring protection by a rectal microbicide is much larger than that of the vagina since it is an open cavity, and the microbicide must be formulated so there is adequate protection in the areas where infectious virus and virus-infected cells in semen migrate [78]. This also impacts the design of the delivery applicators for rectal gel products. pH is another important consideration in the design of vaginal and rectal microbicides. The formulation for each product needs to take into account the differences in the pH of the compartment with vaginal pH of approximately 4.5 and a neutral rectal pH [83]. This pH discordance between the compartments could become inconsequential by use of formulations with minimal buffer capacity causing a shift in pH to match the pH of the local fluids. Since results obtained in the tenofovir trial [82] using the vaginal-optimized gel yielded some adverse reactions when used rectally, the gel was reformulated and is now being evaluated as part of MTN-007. *In vitro* and *ex vivo* data indicated that this gel was more suitable for the rectal environment [84]. Physiologically it appears that prevention success will require different gels for different compartments, although it is evident that the formulation of a product for dual compartment use would be most practical and acceptable given the sexual practices of the users embracing both vaginal and anal intercourse during the same sexual encounter. Development of this type of product will require careful consideration and design so that the issues of pH, osmolarity, volume, and delivery between the two compartments are addressed.

## 6. Multipurpose Prevention Technologies

Multipurpose prevention technologies have appeared as an important topic of discussion in the microbicide community as an unmet need, but little progress has been made in the development and advancement of such a product. Prevention strategies have mostly focused individually on prevention of unplanned pregnancy, prevention of other reproductive tract infections, and prevention of STIs. However, accumulating data indicate that these issues are linked, suggesting that a woman at risk for pregnancy is also at risk for contracting an STI or other reproductive tract infection [85]. Additionally, a certain stigma is associated with self-identifying as “high-risk” for HIV and STI, and most women are reluctant to do so, even those that are truly at high risk. Linking pregnancy prevention with disease prevention with a single product could aid in the motivation for women to actively obtain and utilize microbicide products. This substantiates the need for Multipurpose prevention strategies that might include combinations of agents that would target prevention of pregnancy and HIV, pregnancy and other STIs, HIV and other STIs and pregnancy, and HIV and other STIs. These products would need to be affordable, acceptable, and easy to use. Significant research on dual-purpose protection technologies that include vaginal spermicidal anti-infective agents and physical barrier devices has been performed [86, 87]. Anti-infective and contraceptive microbicides could be developed in three ways based on utilizing a single drug with dual activity, a combination of a microbicidal compound with a contraceptive agent or a combination of a drug with a device. Relevant technologies do exist including male and female condoms to prevent pregnancy and STIs but they are not widely accepted [85, 88]. Recent advances in microbicide development have also provided a foundation for the development of other Multipurpose prevention products, including multipurpose IVRs and diaphragms with contraceptives and anti-STI microbicides [85, 89] and probiotics to treat and deliver drugs [85, 90], a successful product will likely be dependent on scientific innovation and persistence and will require a concerted scientific and financial effort between many organizations. A product of this nature could have substantial implications on the well-being of women and men throughout the developed and developing world.

## 7. Microbicide Acceptability and User Perception

Although a variety of studies have addressed the issue of microbicide acceptability and user perception, product acceptability to the end user remains one of the most critical parameters in developing a successful microbicide product. A product with undesirable characteristics will ultimately result in poor adherence, poor PK, and poor efficacy. Morrow and Hendrix have described linkages between acceptability, PK, and toxicity and how each can greatly impact the other [91]. For this reason evaluation of these microbicide properties is now linked in human clinical trials. Thus, acceptability studies are rather well accepted and utilized in the microbicide field and are not identified as a gap in current product development. However the importance of factoring acceptability into each of the gap evaluations described above remains a critical component of microbicide development algorithms and should be addressed early in development. Our current research has involved a better understanding of the user perceptions of different dosing volumes and delivery vehicles on microbicide product acceptability, as well as understanding user's desire for products which have dual compartment use (manuscripts in press). 

## 8. Summary

Great strides have been made in the development of microbicide products to prevent the sexual transmission of HIV. The microbicide development field has exploited strides in the understanding of mechanisms of HIV transmission, which has resulted in better designed and more predictive assays and models to assess the efficacy and toxicity of candidate products. However, significant gaps in understanding still exist which must be better defined and understood in order for the field to define and prioritize new products for clinical evaluation and eventual use as microbicide products. In order for a microbicide to be successful it will need to be the right product in the right place, at the right time, and at the right concentration. Therefore we need to understand how and where HIV infects target cells in the vagina or rectum, the role of cell-free versus cell-associated virus in initial infectious events, and how and where the virus disseminates after initial infection. The vagina and rectum need to be well understood, and microbicide activity needs to be evaluated in the context of these environments early in preclinical development. Better *in vitro* and *ex vivo* assays need to be developed to address the issues of PK and PD as a means to predict the dosing that will be required for a product in clinical trial. Critical to the dosing requirement are the formulation and delivery of the active pharmaceutical ingredient. Finally, a product will only be successful if it is going to be used, and thus the product needs to be acceptable to the end user. The future of microbicides resides in developing products that will work in both the rectum and vagina and those that are multiprevention agents. As the microbicide field evolves, the preclinical assays and models must adapt to the knowledge obtained from successful and failed clinical trials and development programs. In addition, the limitations of these preclinical *in vitro* and *ex vivo* assays should be recognized and used in conjunction with animal models so that the most thorough characterization of a microbicide can be achieved ultimately resulting in prioritization of the microbicides with the most potential. This will allow for better product discovery and development through better preclinical and clinical testing algorithms ultimately resulting in better prioritization of products for clinical evaluation.

## Figures and Tables

**Table 1:** Comparison of EC_50_ and EC_99_ values determined in the standard transmission inhibition assay to MTSA defined sterilizing concentration.

Compound	EC_50_ in entry transmission assay	EC_99_ in entry transmission assay	Sterilizing concentration determined in MTSA	
			Experiment 1	Experiment 2
IQP-0528 (*μ*M)	0.017	1.0	0.25	1.25
IQP-0410 (*μ*M)	0.059	1.0	>12.5	>12.5
IQP-1187 (*μ*M)	0.053	1.0	0.02	0.1
AZT (*μ*M)	>0.5	>0.5	>31.25	>31.25
UC781 (*μ*M)	0.009	2.98	0.37	1.9
CV-N (*μ*g/mL)	0.001	0.1	12.5	12.5
Efavirenz (*μ*M)	0.03	0.5	0.05	0.05
Tenofovir (*μ*M)	>10	>10	>97.7	>97.7

The dual acting (entry inhibition and NNRTI) pyrimidinediones IQP-0528, IQP-0410, IQP-1187 [69] nonnucleoside RT inhibitors UC781 and efavirenz, nucleoside RT inhibitor AZT, entry inhibitor cyanovirin-N (CVN), and nucleotide RT inhibitor tenofovir (TFV) were evaluated in the MTSA, and the sterilizing concentration was compared to the EC_50_ and EC_90_ determined in a standard virus transmission assay. The concentrations utilized for each compound in the MTSA were derived from their respective EC_50_ concentrations in a cytopathic effect assay and their TIs (EC_50_/TC_50_). The concentrations which were utilized are as follows: IQP-0528, IQP-0410, and IQP-1187: 10 through 31,250 times the EC_50_ concentration; AZT and UC781: 10 through 31,250 times the EC_50_ concentration; cyanovirin-N: 10 through 6,250 times the EC_50_ concentration; efavirenz: 10 through 31,250 times the EC_50_ concentration; tenofovir: 2.5 through 97.7 times the EC_50_ concentration. All concentrations evaluated represented 5-fold serial increases in drug concentration with the exception of tenofovir which was in 2.5-fold increments. Passages which were positive for virus production were defined by detection of virus in the cell-free supernatant by RT assay. Cells were passaged for 10 passages in the continuous presence of the fixed compound concentration and for an additional 5 passages in the absence of compound. All tested concentrations were significantly below the defined toxic concentration to CEM-SS cells. Passages which were positive for virus production were defined by detection of virus in the cell-free supernatant by RT assay.

The entry assay results used for comparison to the MTSA results were generated from an assay utilizing HeLa-CD4-LTR-*β*-Gal Cells with HIV-1_IIIB_. Compound is added to the preplated cells approximately 15 minutes prior to the addition of virus. Following a 2-hour incubation at 37°/5% CO_2_, residual virus and compound are removed through washing. The culture is incubated for an additional 48 hours at which time compound efficacy is determined by evaluating *β*-galactosidase in the lysate using a chemiluminescent endpoint.
